# Sex differences in the association between insulin resistance and non-fatal myocardial infarction across glycaemic states

**DOI:** 10.1186/s12933-023-02093-y

**Published:** 2024-01-13

**Authors:** Alessia Riccio, Elena Fortin, Linda Mellbin, Anna Norhammar, Per Näsman, Lars Rydén, Giorgio Sesti, Giulia Ferrannini

**Affiliations:** 1 Cardiology Unit, Department of Medicine Solna, Karolinska Institute Stockholm, Stockholm, Sweden; 2 Department of Clinical and Molecular Medicine, Sapienza University of Rome, Rome, Italy; 3 Heart, Vascular and Neuro Theme, Karolinska University Hospital, Stockholm, Sweden; 4 Capio St Görans Hospital, Stockholm, Sweden; 5 Center for safety research, KTH Royal Institute of Technology, Stockholm, Sweden; 6 Internal Medicine Unit, Södertälje hospital, Södertälje, Sweden

**Keywords:** Females, Cardiovascular disease, Prediabetes, HOMA-IR, Visceral adiposity index, Triglycerides/high-density lipoprotein index, PAROKRANK

## Abstract

**Background:**

Females are generally less prone to cardiovascular (CV) events than males, but this protection is trumped by diabetes. The mechanism behind the increased relative risk in females with diabetes is not fully understood. Insulin resistance (IR) is suggested to be a more important contributor to CV morbidity in females than in males. We aim to investigate differences in the association between IR indexes (Homeostatic Model Assessment of IR - HOMA-IR, visceral adiposity index – VAI, and triglycerides/high-density lipoprotein-cholesterol - TG/HDL-C index), and a first non-fatal myocardial infarction (MI) across different glycaemic states.

**Methods:**

IR indexes were calculated in a population with (n = 696) and without (n = 707) a first non-fatal MI, free from known diabetes. MI cases were investigated at least six weeks after the event. All participants were categorized by an oral glucose tolerance test as having normal glucose tolerance, impaired fasting glucose, impaired glucose tolerance, or newly diagnosed diabetes. Comparison of proportion of glycaemic states by sex was tested by chi-square test. The associations between sex, a first non-fatal MI, IR indexes, and traditional CV risk factors were analysed by multivariate logistic regression models. Continuous variables were logarithmically transformed.

**Results:**

Of the total population 19% were females and 81% males, out of whom 47% and 50% had a first non-fatal MI, respectively. Compared with males, females were older, less often smokers, with lower body mass index and higher total cholesterol and high-density lipoprotein cholesterol levels. The proportion of glycaemic states did not differ between the sexes (p = 0.06). Females were less insulin resistant than males, especially among cases and with normal glucose tolerance. In logistic regression models adjusted for major CV risk factors including sex, the associations between VAI and TG/HDL-C index and a first non-fatal MI remained significant only in females (odds ratios and 95% confidence intervals: 1.7, 1.0-2.9, and 1.9, 1.1–3.4 respectively).

**Conclusions:**

These results support the assumption that IR indexes based on anthropometrics and lipid panel, i.e., VAI and TG/HDL-C, could be a better measure of IR and CV-predictor for non-fatal MI in females, even without glycaemic perturbations.

**Supplementary Information:**

The online version contains supplementary material available at 10.1186/s12933-023-02093-y.

## Background

Type 2 diabetes mellitus (T2DM) and its preceding states, impaired fasting glucose (IFG) and impaired glucose tolerance (IGT), are a growing epidemic worldwide [1]. Moreover, patients with T2DM and IGT have a higher risk for future cardiovascular disease (CVD), including coronary artery disease (CAD), than those with normal glucose tolerance (NGT) [2].

T2DM affects cardiovascular (CV) risk differently in females and males. CV events and mortality are usually delayed by approximately ten years in females, presumably due to hormonal differences. On the other hand, females with T2DM have a higher relative risk for fatal and non-fatal CVD than males [3–7]. These differences are possibly due to a more pronounced dysglycaemia, with females progressing earlier from NGT to IGT, a greater accumulation of CV risk factors, and a less efficient management of CV risk factors in females than in males [8, 9]. A possible reason for this deteriorating glucose tolerance may be sex disparities in body composition and insulin resistance (IR). IR is indeed a key factor linking several elements of cardiometabolic dysfunction together [10]. There is some evidence suggesting that the more rapid deterioration of glucose homeostasis in females than in males may be associated with worsening IR due to the greater accumulation of body fat in females [11]. IR can be expressed by various indexes, traditionally derived from measures of plasma glucose and insulin, but few of them are routinely used because of impracticalities [12–15]. Indexes derived from routinely assessed laboratory and anthropometric parameters may be more appropriate in females [13, 16–18].

This study aims to investigate sex differences in the association between IR indexes and a first non-fatal myocardial infarction (MI) across glycaemic states.

## Materials and methods

### Study design and population

The present investigation is a post-hoc analysis based on the population from the Periodontitis and its relation to coronary artery disease (PAROKRANK) study, a multicentre case-control study that recruited subjects with and without a first non-fatal MI from May 2010 to February 2014 at 17 Swedish hospitals. A detailed description of the study has been given previously [19]. Thus, only data of importance for the present investigation is repeated here. Individuals < 75 years old were enrolled during their hospitalization for a first non-fatal acute MI, defined according to international criteria [20]. They were scheduled for follow-up visits 6–10 weeks later at the local department of cardiology. Subjects with previous heart valve replacement, and any condition that could limit their ability to follow the study protocol were excluded. The national population registry was used to identify controls free from prior MI and heart valve replacement but of the same sex, age, and from the same postal code area as the corresponding case.

At the visit casesand controls had been fasting and abstained from smoking for at least 12 h. They were subjected to a physical examination where data about heart rate, blood pressure following five minutes of rest in a sitting position, height, body weight, body mass index (BMI), and waist circumference (WC) were collected. At the same time, a venous blood sample was drawn to analyse the following laboratory values: complete blood count, total cholesterol, high-density lipoprotein cholesterol (HDL-C), triglycerides (TG), creatinine, fasting plasma glucose (FPG) and glycated haemoglobin A1c (HbA1c) while high-sensitivity C-reactive protein (hsCRP) was analysed on stored samples. Subjects without known T2DM underwent a 2-hour oral glucose tolerance test (OGTT) during which FPG, 30 and 120 min post-load venous-plasma glucose were measured through the point-of-care HemoCue 201 System (HemoCue AB, Ängelholm, Sweden). Plasma was stored for subsequent analysis of fasting plasma insulin using an electro-chemiluminescence immune assay on a COBAS e411 instrument (Roche, Indianapolis, IN, USA) at Pisa’s Metabolism laboratory, in Italy.

Smoking habits were defined as current, previous (stopped more than one month before the visit), or never. Information on previous medical history was based on self-reported data from standardized questionnaires.

For the present analyses, only individualswho underwent an OGTT were included while those with known diabetes were excluded.

### Definitions

Multiple surrogate indexes of IR were calculated according to the formulas derived from glucose, insulin, lipid metabolites, and participants’ anthropometric characteristics.

The *Homeostatic Model Assessment* of IR (HOMA-IR) was defined as fasting insulin (mU/L) × fasting glucose (mmol/L)/22.5 [15].

The *Visceral adiposity index (VAI)* was evaluated with different formulas in females and males [16]:

$$\begin{aligned} &Females: \\ & \left( {{{{\rm{WC}}} \over {36.58\, + \,\left( {1.89\, \times \,BMI} \right)}}\, \times \,\left( {{{TG\,(mmol\,/\,L)} \over {0.81}}} \right)\, \times \,{{1.52} \over {HDL\,(mmol\,/\,L)}}} \right) \\\end{aligned}$$

$$\begin{aligned}&Males: \\ & \left( {{{{\rm{WC}}} \over {39.68\, + \,\left( {1.88\, \times \,BMI} \right)}}\, \times \,\left( {{{TG(mmol\,/\,L)} \over {1.03}}} \right)\, \times \,{{1.31} \over {HDL\,(mmol\,/\,L)}}} \right) \\ \end{aligned}$$

The *triglycerides/HDL-C (TG/HDL-C)* index was determined as the ratio between triglycerides (mmol/L) and HDL-C (mmol/L) [18, 21].

The TyG index was calculated by the following formula:

$$Ln\,{{TG\,(mg\,/\,dL)\, \times \,glucose\,(mg\,/\,dL)} \over 2}$$

Glucose levels obtained during the OGTT were used to classify study participants according to the World Health Organization criteria [22] as having NGT, IFG, IGT, or newly diagnosed T2DM.

Estimated glomerular filtration rate (eGFR) was calculated by using the CKD-EPI equation [23]: eGFR = 141 × min(Scr/k, 1)α × max(Scr/k, 1)-1.209 × 0.993Age × 1.018 [if female], where Scr is serum creatinine, k is 0.7 for females and 0.9 for males, α is -0.329 for females and − 0.411 for males, min indicates the minimum of Scr/k or 1, and max indicates the maximum of Scr/k or 1.

### Statistical analyses

Continuous variables are expressed as median and interquartile range (IQR) and compared by the Mann-Whitney test while categorical variables are reported as numbers and proportions and compared by chi-square statistics.

Age, BMI, hsCRP, total cholesterol, TG, HDL-C, HOMA-IR, VAI, and TG/HDL-C were natural log-transformed for statistical analyses.

The independent association between sex, IR indexes, a first non-fatal MI, and the following CV risk factors: age, BMI, hsCRP, known family history of CVD, smoking habit, glycaemic status, and blood lipids were investigated by fitting a multivariable regression model. An interaction term between sex and the investigated IR index was inserted to assess whether there was any effect modification of these associations according to sex. The multivariable models were run separately in females and males for indexes where the interaction term was significant (p ≤ 0.05).

Variables that are incorporated into the formulas of each index were not computed in the regression models due to potential collinearity. Multicollinearity between variables comprised in the multiple logistic regression models was assessed by the variance inflection factor.

A two-sided p-value ≤ 0.05 was considered statistically significant. All statistical analyses were performed by SPSS software program version 27 for Windows (IBM CORP, Armonk, NY, USA).

## Results

**Fig. 1:**
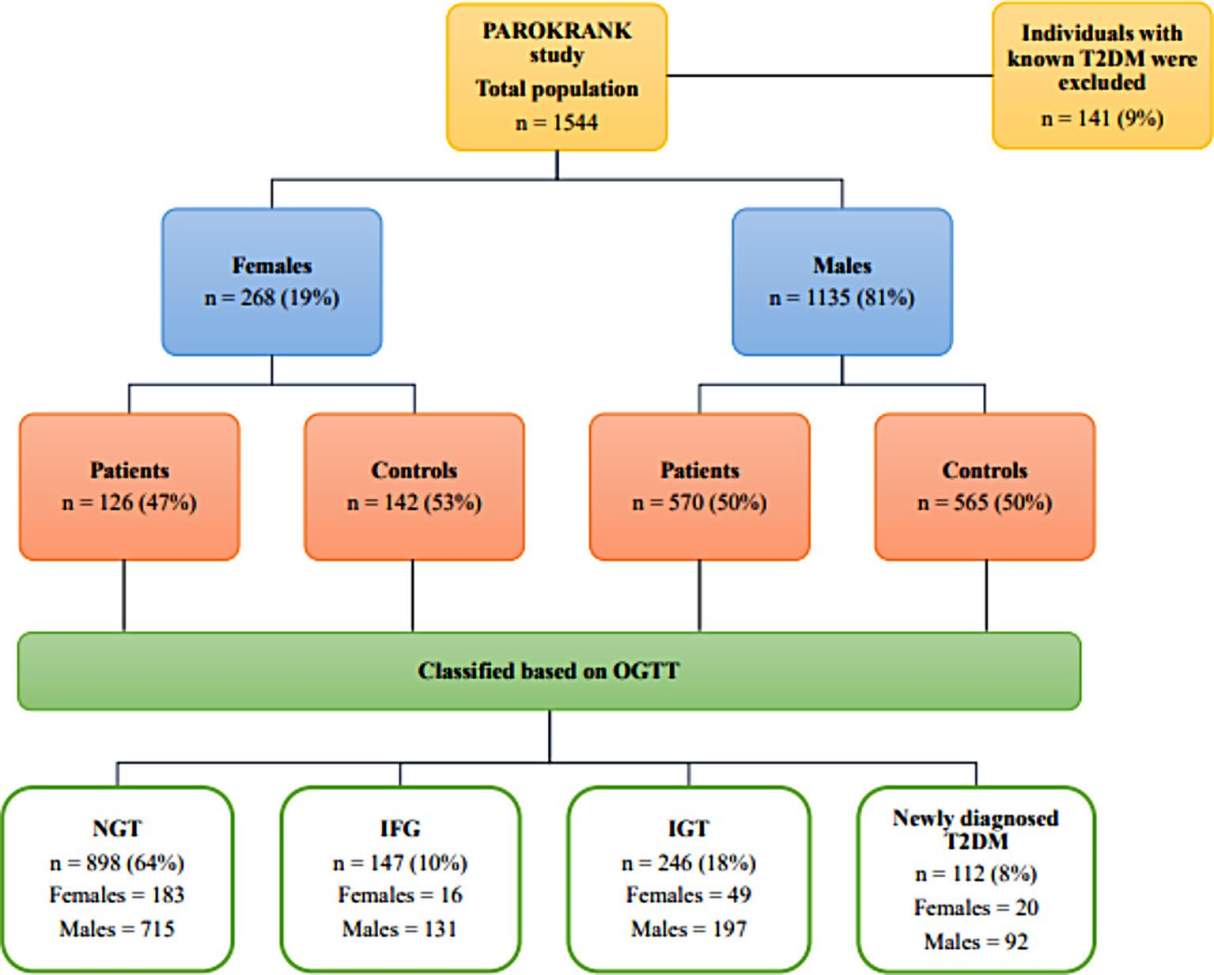
Flow chart of study population divided by sex, presence of first non-fatal myocardial infarction, and glycaemic states defined according to OGTT. IFG = impaired fasting glucose; IGT = impaired glucose tolerance; NGT = normal glucose tolerance; OGTT = oral glucose tolerance test; T2DM = type 2 diabetes mellitus

The study population comprises 1403 participants of whom 268 (19%) were females and 1135 (81%) males. A total of 126 (47%) females and 570 (50%) males had a first non-fatal MI, respectively (Fig. 1). The baseline anthropometric and metabolic characteristics of the population are shown in Table 1. Compared with males, females were older, less often smokers, with a lower BMI and WC, and had lower triglycerides, fasting plasma glucose, and insulin concentrations, but higher levels of total cholesterol and HDL-C. Generally, females were less insulin resistant than males, as they had statistically significant lower HOMA-IR, VAI, TG/HDL-C and TyG levels (Table 1). No differences between the two sexes were observed in the proportion of different glycaemic states (p = 0.06). These differences in anthropometric and metabolic characteristics between the two sexes were maintained even within the different glycaemic states (Supplemental Table 1).

**Table 1:** Baseline characteristics of the whole population, reported as number (%) or median (interquartile range)

	Females 268 (19)	Males 1135 (81)	P value (F vs. M)
Age (years)	65 (60–69)	63 (57–67)	< 0.001
Smoking, yes	126 (48)	559 (50)	0.005
Index MI, yes	126 (47)	570 (50)	0.35
Glycaemic states	-	-	0.06
NGT	183 (68)	715 (63)	0.77
IFG	16 (6)	131 (12)	5.20
IGT	49 (18)	197 (17)	0.09
Newly detected T2DM	20 (8)	92 (8)	0.09
**Medical History**			
Hypertension, yes	84 (32)	343 (30)	0.66
Known family history of CVD*, yes	95 (35)	336 (30)	0.18
**Anthropometrics - vitals**			
BMI (kg/m^2^)	25.7 (23–29)	26.6 (24.5–28.9)	0.001
Obesity, yes	52 (20)	194 (17)	0.36
Waist circumference (cm)	90 (83–100)	100 (93–106)	< 0.001
Systolic blood pressure (mmHg)	133 (120–145)	130 (120–140)	0.20
Diastolic blood pressure (mmHg)	80 (70–86)	80 (74–87)	0.05
Heart rate (bpm)	72 (60–83)	72 (63–85)	0.21
**Laboratory**			
Total cholesterol (mmol/L)	5 (4–6)	4.6 (3.7–5.6)	< 0.001
HDL-C (mmol/L)	1.6 (1.3–1.9)	1.2 (1.1–1.5)	< 0.001
Triglycerides (mmol/L)	1.1 (0.8–1.4)	1.2 (0.9–1.6)	0.005
hsCRP (mg/L)	1.4 (0.7-3)	1.3 (0.7–2.4)	0.07
Creatinine (µmol/L)	68 (60–74)	83 (76–92)	< 0.001
eGFR (mL/min/1.73m^2^)	82.9 (73.6–93.2)	86.7 (76.3–94.2)	0.009
Fasting plasma glucose (mmol/L)	5.3 (4.9–5.8)	5.6 (5.1–6.1)	< 0.001
2 h post-load glucose (mmol/L)	6 (4.9–7.8)	6.2 (5-7.6)	0.72
HbA1c (mmol/mol)	38 (35.5–41)	38 (35–41)	0.28
Fasting plasma insulin (mU/L)	9.8 (7.1–13.5)	11 (7.6–16.6)	< 0.001
**Insulin resistance indexes**			
HOMA-IR	2.3 (1.6–3.3)	2.7 (1.8–4.3)	< 0.001
VAI	0.9 (0.6–1.4)	1.3 (0.9–1.9)	< 0.001
TG/HDL-C	1.5 (1.1–2.4)	2.1 (1.4–3.2)	< 0.001
TyG	8.4 (8.1–8.7)	8.5 (8.2–8.9)	< 0.001
**Pharmacological treatment**			
Beta-blockers, yes	131 (49)	576 (51)	0.58
Renin-angiotensin inhibitors, yes	125 (47)	613 (55)	0.03
Calcium-antagonist, yes	30 (11)	124 (11)	0.90
Diuretics, yes	24 (9)	85 (8)	0.42
Statins, yes	137 (51)	621 (55)	0.28

Continuous variables were expressed as median (IQR) and compared by Mann-Whitney test; categorical variables were reported as numbers and proportions and compared by chi-square test

*Close relative with CVD at < 60 years of age

BMI = body mass index; CVD = cardiovascular disease; eGFR = estimated glomerular filtration rate; HbA1c = glycol-haemoglobin A1c; HDL-C = high-density lipoprotein cholesterol; HOMA-IR = homeostasis model assessment-insulin resistance; hsCRP = high-density C-reactive protein; IFG = impaired fasting glucose; IGT = impaired glucose tolerance; MI = myocardial infarction; NGT = normal glucose tolerance; T2DM = type 2 diabetes mellitus; TG = triglycerides; TyG = triglycerides x fasting glucose; VAI = visceral adiposity index

Table 2 shows the IR indexes in casesand controls according to sex, in the whole population, and within each glycaemic state. In the overall population, among both cases and controls, females were less insulin resistant than males. These sex difference persisted in the NGT group, with VAI, TG/HDL-C index and TyG remaining significantly different between females and males in both cases and controls. In the IFG subgroups, VAI and TG/HDL-C index were significantly different between females and males in casesonly, whereas in the IGT class this difference persisted also in controls. In individuals with newly diagnosed T2DM there were no sex differences in VAI, TG/HDL-C and TyG but higher HOMA-IR in male cases.

**Table 2:** Insulin resistance indexes in patients and controls in the whole population and among the glycaemic states

	**Females**			**Males**				
	**Cases** **126 (47)**	**Controls** **142 (53)**	**P value**	**Cases 570 (50)**	**Controls** **565 (50)**	**P value**	**P value** **cases F vs M**	**P value controls** **F vs M**
HOMA-IR	2.59 (1.87–3.51)	2.06 (1.43–3.04)	0.001	3.03 (2.09–4.53)	2.54 (1.60–3.87)	< 0.001	0.002	0.001
VAI	0.91 (0.69–1.47)	0.82 (0.53–1.26)	0.01	1.41 (0.98–1.98)	1.25 (0.79–1.95)	< 0.001	< 0.001	< 0.001
TG/HDL-C	1.66 (1.20–2.63)	1.39 (0.93–2.12)	0.009	2.28 (1.59–3.23)	2.00 (1.28–3.11)	< 0.001	< 0.001	< 0.001
TyG	8.44 (8.19–8.72)	8.38 (8.09–8.76)	0.15	8.54 (8.28–8.86)	8.53 (8.23–8.92)	0.63	0.02	< 0.001
**NGT**								
	**Females**			**Males**				
	**Cases** **70 (38)**	**Controls** **113 (62)**	**P value**	**Cases** **308 (43)**	**Controls** **407 (57)**	**P value**	**P value** **cases** **F vs M**	**P value controls** **F vs M**
HOMA-IR	2.37 (1.68–3.12)	1.94 (1.33–2.53)	0.004	2.54 (1.82–3.87)	2.04 (1.41–3.12)	< 0.001	0.11	0.02
VAI	0.86 (0.62–1.26)	0.73 (0.51–1.21)	0.04	1.36 (0.94–1.89)	1.15 (0.73–1.86)	0.002	< 0.001	< 0.001
TG/HDL-C	1.62 (1.11–2.28)	1.25 (0.90–2.11)	0.03	2.17 (1.56–3.04)	1.90 (1.21–2.95)	0.002	< 0.001	< 0.001
TyG	8.38 (8.08–8.61)	8.33 (8.06–8.67)	0.59	8.51 (8.21–8.77)	8.45 (8.15–8.80)	0.47	0.05	0.02
**IFG**								
	**Females**			**Males**				
	**Cases** **10 (63)**	**Controls** **6 (37)**	**P value**	**Cases** **83 (63)**	**Controls** **48 (37)**	**P value**	**P value** **cases** **F vs M**	**P value controls** **F vs M**
HOMA-IR	3.73 (2.36–4.69)	3.35 (1.88–5.09)	0.71	3.63 (2.67–4.85)	3.75 (2.63–5.89)	0.48	0.82	0.48
VAI	0.82 (0.50–1.27)	0.92 (0.36–1.53)	0.79	1.29 (0.92–1.89)	1.41 (0.98–1.87)	0.62	0.02	0.08
TG/HDL-C	1.37 (0.88–2.13)	1.53 (0.69–2.67)	0.88	2.09 (1.54–3.09)	2.28 (1.63–2.97)	0.60	0.04	0.10
TyG	8.33 (8.22–8.75)	8.60 (7.96–8.94)	0.79	8.54 (8.30–8.87)	8.76 (8.51–9.05)	0.02	0.29	0.22
**IGT**								
	**Females**			**Males**				
	**Cases** **33 (67)**	**Controls** **16 (33)**	**P value**	**Cases** **120 (61)**	**Controls** **77 (39)**	**P value**	**P value** **cases** **F vs M**	**P value controls** **F vs M**
HOMA-IR	3.00 (2.14–3.65)	3.33 (2.38–4.44)	0.22	3.41 (2.33–5.03)	3.82 (2.43–5.38)	0.26	0.06	0.43
VAI	1.03 (0.75–1.53)	1.01 (0.63–1.17)	0.35	1.55 (1.11–2.54)	1.45 (0.95–2.58)	0.67	< 0.001	0.005
TG/HDL-C	1.82 (1.35–2.75)	1.69 (1.04–2.04)	0.23	2.51 (1.81–3.99)	2.28 (1.54–4.27)	0.63	0.01	0.01
TyG	8.51 (8.29–8.79)	8.46 (8.24–8.85)	0.57	8.63 (8.38–8.96)	8.69 (8.44–9.21)	0.17	0.14	0.03
**Newly diagnosed T2DM**								
	**Females**			**Males**				
	**Cases** **13 (65)**	**Controls** **7 (35)**	**P value**	**Cases** **59 (64)**	**Controls** **33 (36)**	**P value**	**P value** **cases** **F vs M**	**P value controls** **F vs M**
HOMA-IR	2.70 (2.12–4.25)	4.51 (2.49–5.55)	0.14	5.12 (2.83–7.57)	5.79 (3.43–7.53)	0.50	0.02	0.53
VAI	0.99 (0.73–1.92)	1.26 (0.96–1.98)	0.37	1.49 (1.01–2.01)	1.28 (0.86–2.31)	0.91	0.17	0.89
TG/HDL-C	1.75 (1.28–3.22)	2.22 (1.77–3.27)	0.39	2.47 (1.57–3.26)	2.11 (1.37–3.63)	0.86	0.32	0.94
TyG	8.76 (8.49–9.11)	8.84 (8.72–8.95)	0.54	8.68 (8.39-9.00)	8.92 (8.53–9.43)	0.08	0.87	0.55

Insulin resistance indexes were expressed as median (IQR) and compared by the Mann-Whitney test

HDL-C = high-density lipoprotein cholesterol; HOMA-IR = homeostasis model assessment-insulin resistance; IFG = impaired fasting glucose; IGT = impaired glucose tolerance; NGT = normal glucose tolerance; T2DM = type 2 diabetes mellitus; TG = triglycerides; TyG = triglycerides x fasting glucose; VAI = visceral adiposity index

At multivariable logistic regression analysis, the interaction term between the IR indexes and sex was statistically significant for VAI and TG/HDL-C index (Supplemental Table 2), thus multivariable logistic regression models were run separately in females and males (Table 3). The univariate association between HOMA-IR, VAI and TG/HDL-C and a first non-fatal acute MI, separately, was significant both in females and males. In the multivariate models the associations between VAI and TG/HDL-C index, separately, and a first non-fatal acute MI was significant in females (VAI: OR 1.7, 95% CI 1.0-2.9; TG/HDL-C index: OR 1.9, 95% CI 1.1–3.4) but not in males (VAI: OR 1.2, 95% CI 0.9–1.5; TG/HDL-C index: OR 1.2, 95% CI 0.9–1.5). Vice versa, the association between HOMA-IR, TyG and a first non-fatal MI was significant in males (HOMA-IR: OR 1.4, 95% CI 1.0-1.9; TyG: OR 0.5, 95% CI 0.3–0.6) but not in females (HOMA-IR: OR 1.5, 95% CI 0.8–2.9; TyG: OR 0.7, 95% CI 0.3–1.4).

**Table 3:** **Multivariate analysis between non-fatal acute myocardial infarction and several risk factors for cardiovascular disease in females and males**

	Dependent variable: non-fatal acute MI		
	IR index	OR (95% CI) in females	OR (95% CI) in males
	HOMA-IR	1.8 (1.2–2.8)	1.7 (1.4–2.1)
**Model 1** includes age, BMI, smoking habit, known family history of CVD, hsCRP, glycaemic state, triglycerides, HDL-cholesterol and **HOMA-IR**		1.5 (0.8–2.9)	1.4 (1.0-1.9)
	VAI	1.8 (1.2–2.7)	1.3 (1.1–1.5)
**Model 2** includes age, smoking habit, known family history of CVD, hsCRP, glycaemic states and **VAI**		1.7 (1.0-2.9)	1.2 (0.9–1.5)
	TG/HDL-C index	1.9 (1.2–2.9)	1.3 (1.1–1.5)
**Model 3** includes age, BMI, smoking habit, known family history of CVD, hsCRP, glycaemic states and **TG/HDL-C index**		1.9 (1.1–3.4)	1.2 (0.9–1.5)
	TyG	1.6 (0.9–2.8)	0.9 (0.8–1.3)
**Model 4** includes age, BMI, smoking habit, known family history of CVD, hsCRP, glycaemic states, HDL-choleserol and **TyG**		0.7 (0.3–1.4)	0.5 (0.3–0.6)

Continuous variables were natural log transformed

BMI = body mass index; CI = confidence interval; CVD = cardiovascular disease; HOMA-IR = homeostasis model assessment-insulin resistance; hsCRP = high sensitivity C-reactive protein; IR = insulin resistance; MI = myocardial infarction; OR = odds ratio; TG/HDL-C = triglycerides/high-density lipoprotein; TyG = triglycerides x fasting glucose; VAI = visceral adiposity index

## Discussion

In this post-hoc analysis two surrogate indexes of IR, containing metabolic and anthropometric parameters, namely VAI and TG/HDL-C index, were significantly associated with a first MI in females, but not in males, independently of traditional CV risk factors and glycaemic state, the latter characterized by OGTT.

In the present population, there were sex differences in anthropometric and metabolic characteristics. Females displayed a better cardio-metabolic risk profile than males since they were less often smokers, had lower BMI, WC and triglycerides levels, and were less insulin resistant than males. In general, female sex is characterized by the storage of adipose tissue in subcutaneous sites as compared with preferential visceral deposition in males [24]. Previous studies have shown that in a population with normal blood glucose levels females are more insulin sensitive than males [25], although this sex advantage disappears in females with diabetes [26]. Additionally, the VIRGO study found that young females with MI only had a slightly more favourable lipid panel compared with males, suggesting that the sex difference in outcomes after MI cannot be explained by dyslipidaemia only [27]. Several clamp studies, investigating how sex can affect insulin sensitivity in individuals with NGT, showed higher insulin-stimulated glucose disposal in females than in males [28–36]. In our comparison, females displayed lower fasting plasma glucose and insulin levels, and consequently, lower HOMA-IR than their male counterparts. This is in accordance with previous work showing a higher prevalence of IFG in males than in females [8]. However, HOMA-IR might not be the most accurate way to express IR in females, considering that it mainly represents hepatic IR, whereas females would be more exposed to peripheral IR [37]. Using indexes that include anthropometric characteristics and lipids could help clarify the mechanisms underlying the sex differences across glycaemic states.

Another important finding is that, in both sexes, individuals with a first MI had consistently higher values of all IR indexes compared with controls, not only in the general study population but also in the subgroup with NGT. This is remarkable, as we classified NGT quite strictly, excluding not only subjectswith IGT and T2DM but also those with IFG. This, together with the fact that cases, all surviving a first MI, were relatively healthy, further supports the hypothesis that a certain degree of metabolic derangement exists in CAD, even without glycaemic perturbations [38].

Thus, even if VAI and TG/HDL-C index were lower in females than in males, they might capture an early stage of metabolic disturbance that is not pictured by the glycaemic state but is still clinically important and they could be better predictors of MI. This is in line with a recent study highlighting that VAI is associated with CV events in normal weight and over-weight subjects, but not in those who had obesity [39], suggesting that indexes derived from multiple parameters, both anthropometric and laboratory, are more able to identify CV risk in healthier populations.

Additionally, a Chinese study shows that VAI is significantly associated with intracranial atherosclerotic stenosis in middle-aged and elderly females [40]. Accordingly, in a recent clamp-based study in subjectswithout diabetes, there was greater deterioration of insulin sensitivity and greater fat accumulation in females than in males [11]. Therefore, we hypothesized that VAI and TG/HDL-C index could have a better performance than OGTT-derived glycaemic status and HOMA-IR in identifying clinically meaningful IR, with the advantage that they are more practical than OGTT or clamps. Indeed, both VAI and TG/HDL-C index are based on anthropometrics and lipid profiles, (and do not at all include insulin levels), which are routinely assessed, and they are quite well validated.

Assessing VAI and TG/HDL index in females could contribute to the early identification of metabolic deterioration and to instituting preventive measures, such as lifestyle modifications, that could prevent them from developing T2DM and hence losing advantages in terms of CV risk. Indeed, with CV risk stratification being widely based on the limited number of traditional risk factors derived from the Framingham study, our findings provide a rationale for further exploring the possibility of finding risk factors that are more specific for CV risk in females.

The INTERHEART study tried to widen the view on CV risk factors but confirmed that the two most important ones were smoking and increased lipids, followed by hypertension and diabetes, and the importance of all CV risk factors was similar in both sexes, regardless of age and geographical region [41]. Although females remain less represented in CV clinical trials than men, recent years have brought a better understanding of the sex differences in the biological processes accounting for CV risk factors, allowing for an expansion of the number of factors that might play a key role in CV risk. The main challenge in the following years will be to more equitably include both sexes in trials, aiming at adopting efficient preventive measures in females and males, separately.

## Strengths and limitations

The present study has several strengths. Firstly, the study cohort was recruited from 17 Swedish hospitals, covering a nationwide geographical area and various educational and socioeconomic states. The whole population is well characterised and relatively young and healthy, as it includes only cases with a first MI and a well-matched control population. Some limitations should be underlined. As a post-hoc investigation it can only be hypothesis-generating and the present findings need further confirmation. The proportion of females in the complete population (19%) was relatively low and might restrict the power of the analyses. However, this proportion is in line with epidemiological data for a MI population with an upper age limit of 75 years reported in SWEDEHEART during the time of the recruitment, further considering two aspects: we excluded individuals in the first six to ten weeks after the acute event, when females have worse outcomes compared with males, and those with known diabetes, where the proportion of females is higher. Additionally, IR was not evaluated with the gold standard method, the Euglycaemic Hyperinsulinemic Clamp, but with surrogate indexes, however validated and more clinically practical.

## Conclusion

IR might be of special importance as a CV risk factor in females. In particular, IR indexes based on anthropometrics and a lipid panel, i.e., VAI and TG/HDL-C index, may contribute to CV risk stratification in females, independently of their glycaemic state. Further studies are needed to assess the prognostic value of these indexes.

### Electronic supplementary material

Below is the link to the electronic supplementary material.

**Supplementary Material 1:** Supplemental Table 1. Main baseline characteristics within glycaemic states

**Supplementary Material 2:** Supplemental Table 2. Multivariate analysis between sex and several risk factors for cardiovascular disease

## Data Availability

The datasets used during the current study are available from L.R. or G.F. on reasonable request.

## List of abbreviations

BMI: body mass index
CAD: coronary artery disease
CI: confidence interval
CV: cardiovascular
CVD: cardiovascular disease
eGFR: estimated glomerular filtration rate
FPG: fasting plasma glucose
HbA1c: glycated haemoglobin A1c
HDL-C: high-density lipoprotein-cholesterol
HOMA-IR: Homeostatic Model Assessment of insulin resistance
hsCRP: high-sensitivity C-reactive protein
IFG: impaired fasting glucose
IGT: impaired glucose tolerance
IQR: interquartile range
IR: insulin resistance
MI: myocardial infarction
NGT: normal glucose tolerance
OGTT: oral glucose tolerance test
OR: Odds ratio
PAROKRANK: Periodontitis and its relation to coronary artery disease
Scr: serum creatinine
T2DM: type 2 diabetes mellitus
TG: triglycerides
TG/HDL-C: triglycerides/high-density lipoprotein-cholesterol
TyG: triglycerides x fasting glucose
VAI: visceral adiposity index
WC: waist circumference
