# Supplementary material for: Sex differences in the association between insulin resistance and non-fatal myocardial infarction across glycaemic states
Source: Cardiovasc Diabetol. 2024 Jan 13;23:25. doi: 10.1186/s12933-023-02093-y (PMC10787422; doi:10.1186/s12933-023-02093-y)
Supplement: Supplementary file 1 — Supplementary Material 1: Supplemental Table 1. Main baseline characteristics within glycaemic states [file 12933_2023_2093_MOESM1_ESM.docx]

**Supplemental Table 2**. **Multivariate analysis between sex and several risk factors for cardiovascular disease.**

|  | **Dependent variable: sex** | | |
| --- | --- | --- | --- |
|  | **Covariates** | **OR (95% CI)** | **p value** |
| **Model 1** includes age, BMI, hsCRP, smoking habit, known family history of CVD, glycaemic states, triglycerides, HDL-C and first non-fatal acute MI | Age  BMI  hsCRP  Known family history of CVD  Smoking habit (no smokers)  Smoking habit (current smokers)  Smoking habit (ex smokers)  NGT  IFG  IGT  New Diagnosis T2DM  Triglycerides  HDL-C  First non-fatal acute MI | 2.8 (0.7-10.4)  0.6 (0.2-2.2)  1.3 (1.1-1.5)  1.4 (1.0-2.0)  -  0.9 (0.7-1.3)  1.9 (1.1-3.2)  -  0.5 (0.2-0.9)  1.1 (0.7-1.7)  0.85 (0.5-1.6)  1.1 (0.7-1.6)  31.5 (15.8-64.2)  1.6 (1.1-2.2) | 0.13  0.48  0.002  0.04  0.02  0.68  0.02  0.12  0.02  0.70  0.61  0.80  <0.001  0.01 |
| **Model 2** includes age, BMI, hsCRP, known family history of CVD, smoking habit, 2-hour post-load glucose, total cholesterol, first non-fatal acute MI and HOMA-IR | Age  BMI  hsCRP  Known family history of CVD  Smoking habit (no smokers)  Smoking habit (current smokers)  Smoking habit (ex smokers)  NGT  IFG  IGT  New Diagnosis T2DM  Triglycerides  HDL-C  First non-fatal acute MI  HOMA-IR | 2.6 (0.7-10.1)  0.8 (0.2-2.9)  1.3 (1.1-1.5)  1.4 (1.0-1.9)  -  0.9 (0.7-1.3)  1.9 (1.1-3.2)  -  0.5 (0.2-0.9)  1.1 (0.7-1.8)  0.9 (0.5-1.7)  1.1 (0.7- 1.6)  31.3 (15.5-63.3)  1.6 (1.1-2.2)  0.9 (0.6-1.2) | 0.15  0.71  0.002  0.05  0.03  0.70  0.02  0.16  0.04  0.57  0.78  0.70  <0.001  0.01  0.45 |
| **Model 3** includes age, hsCRP, known family history of CVD, smoking habit, glycaemic states, first non-fatal acute MI and VAI | Age  hsCRP  Known family history of CVD  Smoking habit (no smokers)  Smoking habit (current smokers)  Smoking habit (ex smokers)  NGT  IFG  IGT  New diagnosis T2DM  First non-fatal acute MI  VAI  Interaction p VAI#sex | 4.7 (1.3-16.5)  1.3 (1.1-1.5)  1.5 (1.1-2.1)  -  1.1 (0.8-1.5)  2.3 (1.4-3.8)  -  0.4 (0.2-0.8)  1.1 (0.7-1.7)  0.8 (0.5-1.5)  1.1 (0.8-1.5)  0.3 (0.2-0.4)  - | 0.02  0.002  0.02  0.005  0.61  0.001  0.07  0.01  0.62  0.52  0.60  <0.001  **0.018** |
| **Model 4** includes age, BMI, hsCRP, smoking habit, known family history of CVD, glycaemic states, first non-fatal acute MI and TG/HDL-C index | Age  BMI  hsCRP  Known family history of CVD  Smoking habit (no smokers)  Smoking habit (current smokers)  Smoking habit (ex smokers)  NGT  IFG  IGT  New diagnosis T2DM  First non-fatal acute MI  TG/HDL-C index  Interaction p TG/HDL#sex | 4.9 (1.4-17.5)  0.7 (0.2-2.2)  1.3 (1.1-1.5)  1.5 (1.1-2.0)  -  1.1 (0.8-1.5)  2.1 (1.3-3.5)  -  0.4 (0.2-0.8)  1.1 (0.7-1.6)  0.9 (0.5-1.5)  1.03 (0.8-1.4)  0.4 (0.3-0.5)  - | 0.01  0.49  0.004  0.02  0.01  0.76  0.004  0.07  0.01  0.75  0.62  0.83  <0.001  **0.013** |
| **Model 4** includes age, BMI, hsCRP, smoking habit, known family history of CVD, glycaemic states, HDL-cholesterol, first non-fatal acute MI and TyG | Age  BMI  hsCRP  Known family history of CVD  Smoking habit (no smokers)  Smoking habit (current smokers)  Smoking habit (ex smokers)  NGT  IFG  IGT  New diagnosis T2DM  HDL-cholesterol  First non-fatal acute MI  TyG  Interaction p TyG#sex | 2.8 (0.7-10.4)  0.7 (0.2-2.5)  1.3 (1.1-1.5)  1.4 (1.0-1.9)  -  0.9 (0.7-1.3)  1.9 (1.2-3.3)  -  0.47 (0.2-0.9)  1.1 (0.7-1.7)  0.9 (0.5-1.6)  28.9 (14.4-58.1)  1.6 (1.1-2.2)  0.9 (0.6-1.3)  - | 0.13  0.63  0.001  0.04  0.02  0.74  0.01  0.14  0.03  0.61  0.70  <0.001  0.013  0.48  **0.8** |

Continuous variables were natural log transformed.

Variables incorporated into the formulas of each IR index were not computed in the single regression model of the index to avoid collinearity.

BMI= body mass index; CI= confidence interval; CVD= cardiovascular disease; HOMA-IR= homeostatic model assessment of insulin resistance; hsCRP= high sensitivity C-reactive protein; IFG= impaired fasting glucose; IGT= impaired glucose tolerance; IR= insulin resistance; MI= myocardial infarction; NGT= normal glucose tolerance; OR= odds ratio; T2DM= type 2 diabetes mellitus; TG/HDL-C= triglycerides/high-density lipoprotein cholesterol; TyG= triglycerides x fasting glucose; VAI= visceral adiposity index.
