# Supplementary material for: Sex differences in the association between insulin resistance and non-fatal myocardial infarction across glycaemic states
Source: Cardiovasc Diabetol. 2024 Jan 13;23:25. doi: 10.1186/s12933-023-02093-y (PMC10787422; doi:10.1186/s12933-023-02093-y)
Supplement: Supplementary file 2 — Supplementary Material 2: Supplemental Table 2. Multivariate analysis between sex and several risk factors for cardiovascular disease [file 12933_2023_2093_MOESM2_ESM.docx]

**Supplemental Table 1. Main baseline characteristics within glycaemic states.**

|  | **Females** | **Males** | **P value**  **(F vs M)** |
| --- | --- | --- | --- |
| **NGT** | n=183, 68% | n=715, 63% |  |
| BMI (kg/m^2^) | 25.4 (22.7-28.7) | 26.2 (24.3-28.3) | 0.02 |
| Waist circumference (cm) | 89 (81-98) | 98 (92-104) | <0.001 |
| HDL-C (mmol/L) | 1.6 (1.4-1.9) | 1.3 (1.1-1.5) | <0.001 |
| Triglycerides (mmol/L) | 1 (0.8-1.4) | 1.1 (0.8-1.6) | 0.03 |
| Fasting plasma glucose (mmol/L) | 5.1 (4.7-5.5) | 5.3 (4.9-5.6) | <0.001 |
| 2h post-load glucose (mmol/L) | 5.4 (4.6-6.4) | 5.5 (4.6-6.5) | 0.46 |
| Fasting Plasma Insulin (mU/L) | 8.9 (6.4-12.4) | 9.8 (6.9-14.5) | 0.01 |
| HOMA-IR | 2.03 (1.42-2.88) | 2.29 (1.54-3.40) | 0.002 |
| VAI | 0.77 (0.56-1.26) | 1.27 (0.82-1.86) | <0.001 |
| TG/HDL-C | 1.41 (0.98-2.12) | 2.05 (1.35-3.04) | <0.001 |
| TyG | 8.34 (8.08-8.62) | 8.48 (8.16-8.79) | 0.002 |
| **IFG** | n=16, 6% | n=131, 12% |  |
| BMI (kg/m^2^) | 26 (23.3-28.2) | 27.2 (25-29.4) | 0.13 |
| Waist circumference (cm) | 91 (84-100) | 100 (95-107) | 0.001 |
| HDL-C (mmol/L) | 1.5 (1.3-1.8) | 1.2 (1-1.4) | <0.001 |
| Triglycerides (mmol/L) | 0.9 (0.7-1.4) | 1.1 (0.9-1.5) | 0.08 |
| Fasting plasma glucose (mmol/L) | 6.35 (6.2-6.4) | 6.3 (6.2-6.6) | 0.99 |
| 2h post-load glucose (mmol/L) | 5.6 (5.1-6.4) | 6.1 (5.3-7) | 0.36 |
| Fasting Plasma Insulin (mU/L) | 12.7 (8.3-16.6) | 12.7 (9.5-18.9) | 0.55 |
| HOMA-IR | 3.64 (2.36-4.56) | 3.71 (2.67-5.16) | 0.53 |
| VAI | 0.82 (0.47-1.44) | 1.36 (0.94-1.88) | 0.004 |
| TG/HDL-C | 1.37 (0.87-2.48) | 2.24 (1.55-2.99) | 0.008 |
| TyG | 8.39 (8.20-8.85) | 8.65 (8.40-8.95) | 0.08 |
| **IGT** | n=49, 18% | n=197, 17% |  |
| BMI (kg/m^2^) | 26 (23.5-30.1) | 27.1 (24.9-29.4) | 0.19 |
| Waist circumference (cm) | 90 (85.5-100.5) | 103 (95-108) | <0.001 |
| HDL-C (mmol/L) | 1.4 (1.3-1.8) | 1.2 (1-1.4) | <0.001 |
| Triglycerides (mmol/L) | 1.2 (0.9-1.5) | 1.2 (0.9-1.8) | 0.09 |
| Fasting plasma glucose (mmol/L) | 5.5 (5.1-6.1) | 5.9 (5.5-6.4) | 0.007 |
| 2h post-load glucose (mmol/L) | 8.9 (8.4-9.4) | 8.7 (8.2-9.4) | 0.32 |
| Fasting Plasma Insulin (mU/L) | 11.6 (9-16.1) | 13.7 (9.4-18.9) | 0.10 |
| HOMA-IR | 3.09 (2.22-4.02) | 3.52 (2.38-5.11) | 0.03 |
| VAI | 1.02 (0.75-1.46) | 1.49 (1.04 – 2.55) | <0.001 |
| TG/HDL-C | 1.75 (1.33-2.52) | 2.37 (1.71-4.05) | <0.001 |
| TyG | 8.51 (8.28-8.84) | 8.65 (8.39-9.10) | 0.01 |
| **New Diagnosis T2DM** | n=20, 8% | n=92, 8% |  |
| BMI (kg/m^2^) | 27.2 (23.4-32.1) | 28.1 (24.9-30.9) | 0.48 |
| Waist circumference (cm) | 95 (85-104) | 102 (96-111) | 0.03 |
| HDL-C (mmol/L) | 1.4 (1.2-1.7) | 1.2 (1-1.5) | 0.02 |
| Triglycerides (mmol/L) | 1.2 (1-1.6) | 1.2 (0.8-1.8) | 0.69 |
| Fasting plasma glucose (mmol/L) | 6.7 (5.7-7.2) | 7.1 (6.5-7.5) | 0.01 |
| 2h post-load glucose (mmol/L) | 11.9 (11.1-13.4) | 11.7 (9.7-13.3) | 0.50 |
| Fasting Plasma Insulin (mU/L) | 12.2 (7.5-20.7) | 16.9 (10.5-21.7) | 0.12 |
| HOMA-IR | 3.34 (2.23-5.09) | 5.32 (2.96-7.49) | 0.03 |
| VAI | 1.04 (0.79-1.78) | 1.45 (0.92-2.16) | 0.23 |
| TG/HDL-C | 1.84 (1.34-2.99) | 2.40 (1.44-3.39) | 0.39 |
| TyG | 8.81 (8.60-9.01) | 8.79 (8.41-9.21) | 0.85 |

Continuous variables were expressed as median (IQR) and compared by Mann-Whitney test; categorical variables were reported as numbers and proportions.

BMI= body mass index; HDL-C= high-density lipoprotein cholesterol; HOMA-IR= homeostasis model assessment-insulin resistance; IFG= impaired fasting glucose; IGT= impaired glucose tolerance; NGT= normal glucose tolerance; T2DM= type 2 diabetes mellitus; TG= triglycerides; TyG= triglycerides x fasting glucose; VAI= visceral adiposity index.
